# CYCLOPS: A cyclists’ orientation data acquisition system using RGB camera and inertial measurement units (IMU)

**DOI:** 10.1016/j.ohx.2024.e00534

**Published:** 2024-04-18

**Authors:** Mauricio Arias-Correa, Sebastián Robledo, Mateo Londoño, Johnatan Bañol, Carlos Madrigal-González, John R. Ballesteros, John W. Branch-Bedoya

**Affiliations:** a Vision and Photonics Lab., Engineering Faculty, Instituto Tecnológico Metropolitano., Medellín, Colombia; b Mercado Libre, Colombia; c Universidad Nacional de Colombia, Sede Medellín, Colombia

**Keywords:** RGB camera, Motion capture, Relative orientation, Inertial measurement unit

## Abstract

This paper introduces CYCLOPS, an acquisition system developed to capture images and inertial measurement data of moving cyclists from a vehicle. The development of CYCLOPS addresses the need to acquire useful data for training machine learning models capable of predicting the motion intentions of cyclists on urban roads. Considering its application, it is a completely original development. The system consists of two devices. The first device is installed on the bicycle and is based on an electronic acquisition board comprising an inertial measurement unit (IMU), a microcontroller, and a transceiver for sending the cyclist’s acceleration and orientation data to a vehicle. The second device is installed on the vehicle and uses the same board architecture to acquire the vehicle’s accelerations and orientations, along with an RGB monocular camera. The data is stored in real-time in a laptop’s drive for subsequent analysis and manipulation. The hardware architecture is presented in detail, including the designs to install the devices, for IMUs configuration, and software installation on the laptop. All design and software files required to develop the proposed system are available for download at: doi.org/10.17632/3yx5y8b7tm.1, licensed under the Open-source license CC BY 4.0.

## Graphical abstract

**Figure:**
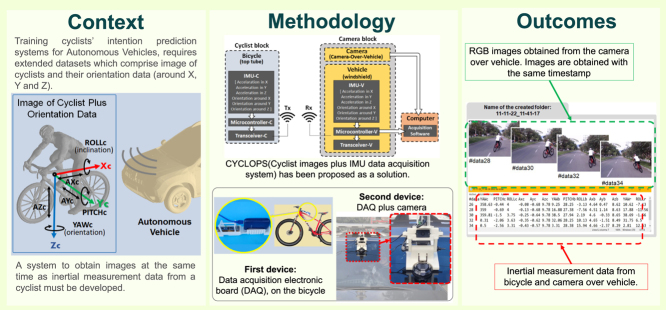

**Specifications table**

**Table:** 

Hardware name	CYCLOPS: Cyclist images plus IMU data acquisition system
Subject area	Engineering
Hardware type	Imaging tools; Field measurements and sensors
Closest commercial analog	No commercial analog is available.
Open source license	CC BY 4.0
Cost of hardware	USD 428.07
Source file repository	https://doi.org/10.17632/3yx5y8b7tm.1

## 1 Hardware in context

Road traffic faces several problems, such as traffic jams, environmental damages associated with human health damages (i.e. air and noise pollution), misused urban space, and road traffic injuries and deaths [1], [2] As stated in [3], road traffic injuries are the 8th leading cause of death for people of all ages (1.35 million in 2016), and more than half are a group comprised of pedestrians, cyclists, and motorcyclists.

Autonomous vehicles (also known as self-driving cars or simply AVs), can operate themselves and perform necessary functions without any human intervention, based on information about their surroundings. Intelligent Transportation Systems (Intelligent Transport Systems or ITS), make use of information and communication technologies, as well as management strategies to provide the users (i.e., AVs, drivers, travelers, pedestrians, cyclists, and motorcyclists), with information that increases their safe and fluent displacement on the roads, through information exchange [1], [4], [5].

Therefore, interaction with other traffic actors (including manually driven vehicles, cyclists, and pedestrians), must be done through effective communication and coordination capabilities between traffic participants [6]. To safely integrate AVs in ITSs, the common human-human interaction between the onboard driver and other traffic participants today must be replaced by an artificial interaction, mainly between the AVs and the Vulnerable Road Users [7], [8], [9].

A Vulnerable Road User (VRU) is a “…road user with an increased risk of being injured or killed in traffic because he is not surrounded by a protective cover which would significantly reduce the severity of an accident”, [10]. This definition covers pedestrians, cyclists, motorcyclists, and people with disabilities or reduced mobility. Pedestrians and cyclists represent 26% of all road traffic deaths [3].

Several research works have been done on vision-based detection systems for VRUs, most of them aiming at pedestrians [11], [12], [13], [14], [15], [16], [17]. Little attention, on the other hand, has been paid to cyclists. In contrast to pedestrian detection, cyclist detection is one of the most challenging environment perception tasks that an AV must face in an ITS scenario [7], [18], mainly due to the cyclists’ visual complexity, variety of possible orientations, aspect ratios and appearance, along with the lack of enough labeled datasets and the presence of occlusions and cluttered backgrounds [19], [20].

Effective training of cyclists’ intention prediction systems is necessary to improve both the perception and performance of the AVs, avoiding - this way, injuries, or deaths. Some researchers have proposed prediction systems based on convolutional neural networks (CNN). In [21] a data-driven approach based on a multilayer perceptron neural network (MLP) is proposed, in conjunction with a polynomial least-squares approximation to forecast the future trajectory of cyclists in a traffic scene observed from a traffic camera. Motion models obtained from a moving vehicle were used for cyclist path prediction on real-world tracks [22]. Two models based on deep stacked recurrent neural networks for the cyclist trajectory prediction task in urban traffic environments were proposed in [23]. The proposed models have shown robust prediction results when evaluated on real-life cyclist trajectory datasets collected using vehicle-based sensors in the urban traffic environment. In [24] start intention is detected with CNN architecture and Motion History Images (MHIs) based on their dataset, the same problem is addressed in [25] training the system with a dataset of 3D poses of cyclists recorded from a moving vehicle in real road traffic. In another context-based path prediction for cyclists [26], authors proposed a Recurrent Neural Network (RNN) to learn the effect of contextual cues directly on the behavior (one related to the actions of the cyclist, one related to the location of the cyclist on the road, and one related to the interaction between the cyclist and the ego-vehicle), then the RNN predicts a Gaussian distribution over the future position of the cyclist.

In every work presented above, an image data set has been used. There exist some open datasets used for cyclist detection, such as the Tshingua–Daimler Cyclist (TDC) dataset presented in [19], the Specialized cyclist detection dataset [27], the KITTI dataset [28], the Waymo open motion dataset [29], and the Lyft dataset [30]. All the mentioned datasets were obtained from moving vehicles on either urban or countryside roads, so that is why they contain information about the cyclist’s context. The orientation detection of a cyclist through CNN-based classifiers is addressed in [31], [32], training the proposed architectures from data sets created by the authors, whose cyclist’s orientation takes value every 45 degrees. However, in none of the cited datasets, the images of the cyclists were correlated with the cyclist’s acceleration and orientation data. This is – mainly – due to the lack of a data acquisition system that allows images of the cyclist to be obtained at the same time as inertial measurement data from an inertial measurement unit (IMU) installed on the camera and from another IMU installed on the bicycle (to correlate these data from the IMUs and obtaining the relative inertial data of the cyclist for each image acquired).

Inertial measurement units have been used as main devices in other works. In [33] is presented the design, assembly, and validation of a small sensor attached to a wearable camera in the operating room to objectively quantify camera motion through an inertial measurement unit coupled to a microcontroller, thus, capturing high-quality intraoperative video of open surgical procedures. The design and validation of a low-cost mobile albedometer that measures the reflection in eight spectral bands in the visible are presented in [34], the system is equipped with a Global Navigation Satellite System (GNSS) receiver, to reference its position and an Inertial Measurement Unit (IMU) to know its absolute orientation, make corrections in real-time or detect errors. A low-cost portable electronic system for estimating step width during the human gait cycle was described in [35] as a supporting device for Performance Oriented Mobility Assessment Test (POMA). The device comprised two sensing nodes and a concentrator. Each sensing node contained a force-sensitive resistor (FSR) inside the subject’s shoe and a time-of-flight camera (TOF) located at the back of their foot. Step width is calculated as the difference between the TOF measurements. After the walk is complete, the information obtained by the FSRs and TOFs is sent via a 433 MHz wireless communication to a personal computer (PC). There is no IMU used in this work, but the data transmission system is like the system that we developed. In [36], a new type of wireless platform designed for probe vehicles in real-time traffic estimation and road surface monitoring is reported. The sensor platform is built around a microcontroller and an Inertial Measurement Unit module. The authors demonstrate some applications of such a platform in smart cities, including trajectory estimation and road condition monitoring. This is the closest application of the same electronic devices that we are using in our work. None of the papers cited above have presented the IMU as a main device in a data acquisition system aimed to obtain a VRU dataset.

**Fig. 1:**
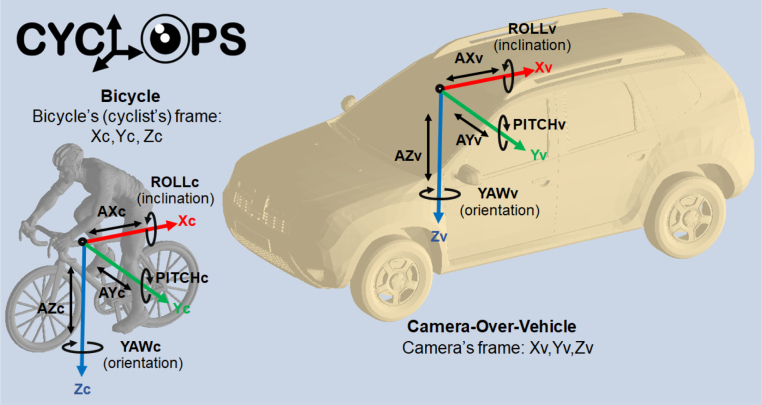
Frame assignment for both, the camera attached to a car’s windshield (over the vehicle) and the bicycle’s top tube (cyclist). For the vehicle, the axes have been named Xv, Yv, and Zv, and rotations around the axis are ROLLv, PITCHv, and YAWv (respectively). Similarly, the frame for the cyclist has axes Xc, Yc, and Zc, and rotations around the axis are ROLLc, PITCHc, and YAWc (respectively). Those frames assignment matches the frames selected for programming both IMUs, the IMU-C for the cyclist and IMU-V for the camera over the vehicle.

**Fig. 2:**
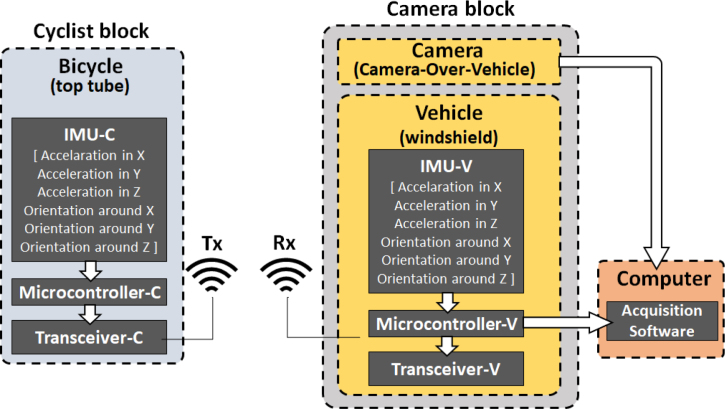
Diagram of the CYCLOPS system. The block Cyclist includes a printed circuit board (PCB-C) attached to the bicycle’s top tube, which comprises an IMU (IMU-C), an Arduino Nano board (Microcontroller-C), and a transceiver HC12 in transmission mode (Transceiver-C) with an antenna. Similarly, the block Camera (the camera is attached to the car’s windshield) is a printed circuit board (PCB) that includes an IMU (IMU-V), an Arduino Nano board (Microcontroller-V), and a transceiver HC12 in reception mode (Transceiver-V) with an antenna. This second block includes the monocular RGB camera. Both, the camera, and the PCB, send data to the executable acquisition software in a computer (laptop).

The contribution of the work presented in this article lies in the development and detailed description of a novel data acquisition prototype named “CYCLOPS” (cyclists’ orientation data acquisition system using RGB camera and inertial measurement units). This prototype consists of two inertial measurement data acquisition devices (the first for a cyclist and the second for a camera attached to a vehicle), and an RGB image acquisition device (the camera). The system acquires as many images as pairs of inertial data (cyclist accelerations and orientations, and camera accelerations and orientations), with a configurable sampling period and total acquisition time. Thus, acceleration and orientation data in the three axes of the cyclist, and the three axes of the camera, are acquired (as seen in Fig. 1). CYCLOPS has been developed to acquire data in urban environments, not in rural settings, therefore the experiments shown in this work validating its performance, have been carried out on paved roads with few irregularities. The overall system is shown in Fig. 2.

For this diagram, the computer is not considered a block, but a commercial device where our own-designed software runs.

## 2 Hardware description

**Fig. 3:**
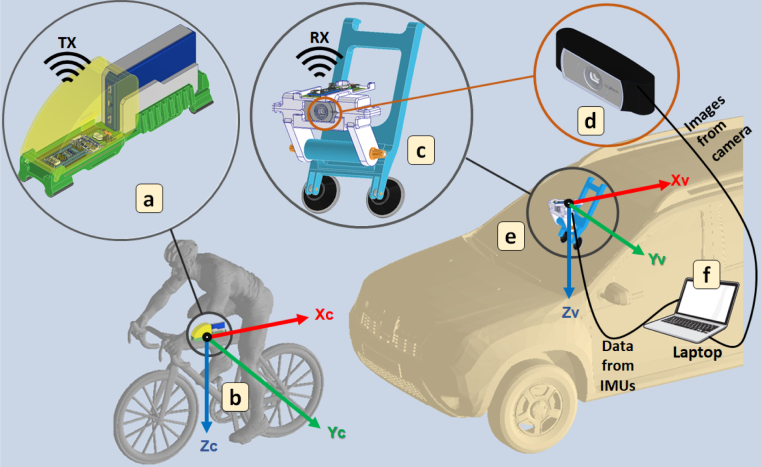
Comprising devices of the proposed system. The first device is enclosed in a housing (a) to be installed on the top tube of the bicycle (b). The data acquired by IMU-C is wirelessly transmitted through Transceiver-C to the second device (c), which includes the camera (d). Using its housing the second device can be attached to the vehicle’s windshield (e). Acquired data are sent to a laptop (f) located inside the vehicle.

The system comprises two inertial measurement data acquisition devices and a monocular RGB camera. The first device is attached to the bicycle’s top tube, and the second device is installed on top of the camera, which in turn has been attached to a vehicle’s windshield. The data acquired by the three devices in each sampling period is sent to a laptop to be structured and stored as inertial measurement data acquired (bicycle and camera over the vehicle), and corresponding RGB images. These devices and their locations can be seen in Fig. 3.

The acquisition time and acquisition period are pre-selected via the acquisition software. The minimum acquisition period is 33.33333 ms, corresponding to the maximum camera acquisition frequency of 30 frames per second (FPS). A detailed description of the structure and components of each device is presented in the following subsections.

### 2.1 Inertial measurement data acquisition devices: first device

**Fig. 4:**
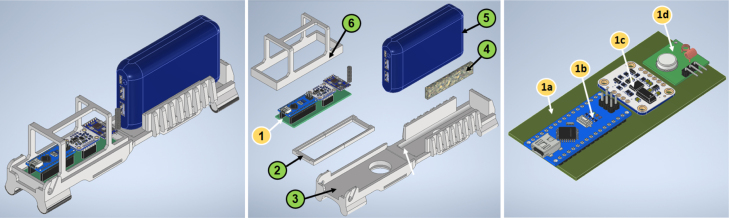
CAD designs for the first device (left). The same device can be seen in an exploded view with numbered parts (center) where (1) is the data acquisition electronic board, (2) is the support frame for the electronic board, (3) is the 3D-printed base, (4) is an adjusting foam pad, (5) is an off-the-shelf power supply device (power bank), and (6) is the 3D-printed bumper. The data acquisition electronic board (right) comprises the PCB over Bakelite substrate (1a), the microcontroller-based board, Arduino Nano in (1b), the Inertial Measurement Unit BNO055 in (1c), and the transceiver HC-12 in (1d).

Both the device installed on the bicycle’s top tube and the second device, which is installed on top of the camera – to form a single unit –, are comprised of the same electronic boards. However, the housings that allow for their installation are different. The similarities and differences between both devices can be better appreciated from Fig. 4, Fig. 8, which show their assembly and exploded views.

The first device consists of an electronic data acquisition board, a commercial power bank to supply power to the electronic devices, and a 3D-printed housing, as shown in Fig. 4. The components are described below.

#### 2.1.1 Data acquisition electronic board

**Fig. 5:**
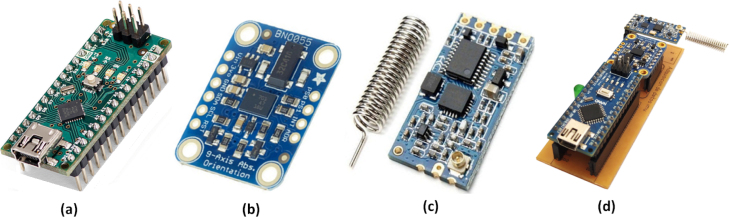
Details of the electronic components used in the acquisition electronic board and their assembly are provided. In (a), the image of the Arduino Nano board is displayed. In (b), the image of the BNO-055 IMU is presented. In (c), the HC-12 transceiver can be observed. The assembly of all the depicted devices on the PCB is shown in (d).

The base of the board is a PCB on a Bakelite substrate, on which the Microcontroller-C (Arduino Nano), the IMU-C (inertial measurement unit), and the Transceiver-C (HC12 transceiver) have been arranged, as shown in Fig. 5. Since the first device is attachable to the bicycle, the microcontroller, IMU, and transceiver will also be referred to as Microcontroller-C, IMU-C, and Transceiver-C respectively, when necessary.

**Microcontroller-based board.** A commercial microcontroller-based board known as the Arduino Nano was used for data processing. As shown in Fig. 5a, this is a small board (43.2 mm × 18 mm) based on the ATmega328 (ATMEL), 5 V power supply, a 16 MHz clock, and uses communication protocols such as UART, I2C, and SPI. It works with a Mini-B USB cable, there are fourteen inputs (D0-D13) with eight analog pins and two reset pins on this board. It also features a 32 KB flash memory. Several hardware-based projects like [37], [38], [39] have successfully used this board. The microcontroller was programmed with the Arduino development environment (Arduino IDE), and we used free Adafruit libraries that are based on the manufacturer’s specifications for data extraction. The algorithm we used for the extraction of the yaw, pitch, roll, x-axis, y-axis, and z-axis acceleration data is customized from these libraries, thus generating a sketch that first reads the calibration data previously stored in the EEPROM memory of the device and assigns it to the IMU, After this reading, a piece of code is executed that is responsible for extracting the desired data, it is also integrated with the HC12 transceiver via UART interface for sending wireless data with target to the vehicle that is pursuing the target where it is joined with the data of this, and the camera tags to generate the desired dataset.

**Inertial measurement Unit (IMU).** Inertial measurement Unit (IMU). For the data acquisition system to fulfill its purpose, the inertial data acquisition board requires an IMU (which has been named IMU-C for the bicycle case and IMU-V for the camera on the vehicle case). An IMU is a device that combines multiple sensors such as accelerometers, gyroscopes, and magnetometers. IMUs are often used in applications where knowing the orientation and motion of an object is important, as seen in [34], [40], [41], [42], [43].

**Fig. 6:**
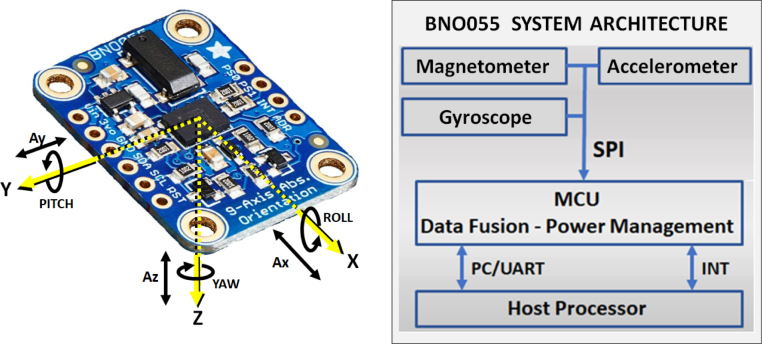
BNO055 absolute orientation sensor. (Left) Axes configuration according to our development requirements, including computed orientation angles delivered by gyroscopes (in Euler angles roll, pitch, and yaw), and accelerations delivered by accelerometers in axes X, Y, and Z. (Right) Basic building blocks (system architecture).

In the data acquisition electronic board of our first device, an IMU BNO055 has been assembled and configured in relative orientation mode. The BNO055 is an intelligent 9-axis absolute orientation sensor that integrates into a single package a triaxial 14-bit accelerometer, a triaxial 16-bit gyroscope (range of ±2000 degrees per second), a triaxial geomagnetic sensor and a 32-bit cortex M0+ microcontroller running Bosch Sensortec sensor fusion software. For optimum system integration, this sensor is equipped with digital bidirectional I2C and UART interfaces as seen in Fig. 6, and the chipsets are integrated into one single 28-pin 3.8 mm × 5.2 mm × 1.1 mm housing [44]. The relative orientation mode of the IMU uses the accelerometer and gyroscope to compute the orientation values around the X, Y, and Z axes (roll, pitch, and yaw, respectively). This is important in our application to know the orientation around the X and Y axes of both the bicycle and the vehicle. The absolute orientation, which is computed relative to the magnetic north using the IMU’s magnetometers and is commonly used for navigation purposes in autonomous vehicles, has been disregarded.

[44].

The IMU also features advanced sensor fusion software (developed by its manufacturer) that aims to fuse the data from the 3 sensors (magnetometer, accelerometer, and gyroscope) with different configuration modes. In our development, we have configured the mode that uses – only – the signals from the accelerometer and gyroscope, providing relative orientation results.

**Transceiver.** To send processed acceleration and orientation data from the first device (in the bicycle) to device 2 (over the camera, in the vehicle), an HC-12 transceiver was used. HC12 is a wireless serial port communication module based on the SI4463 RF chip, has a built-in microcontroller, and can be configured by AT commands, the maximum output power is 100 mW (20 dBm) and the receiver sensitivity differs from 117 dBm to −100 dBm, depending on the baud rate. It accepts 3.2 V–5.5 V and can be used with 3.3 V and 5 V UART voltage devices. Its long-distance wireless transmission (1000 m in open space/5000 bps transmission speed in the air) and working frequency range (433.4–473.0 MHz, up to 100 communication channels), makes this device an optimum choice to assemble in the data acquisition board. The HC12 uses a UART communication interface to receive data (char type) from the Arduino Nano.

**Fig. 7:**
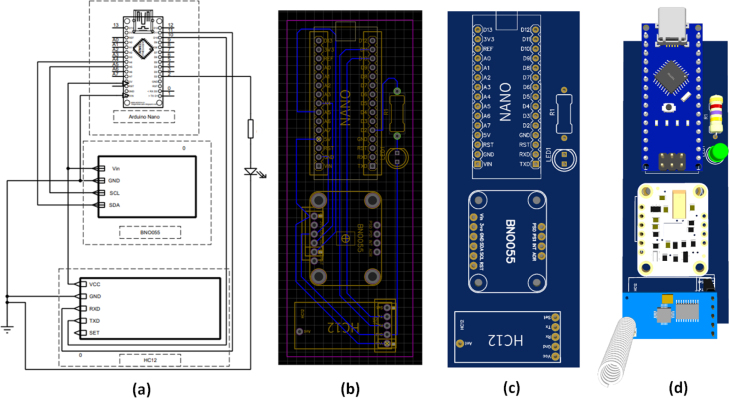
From design to assembly in CAD software, the inertial measurement data acquisition electronic board began with a schematic design (a), then the PCB design was carried out (b and c), and finally a 3D model was designed to understand dimensions and real appearance.

At assembling on the PCB, the Arduino Nano, BNO055, and HC12, the first inertial measurement data acquisition board is obtained. In Fig. 7, the process from design to CAD modeling has been illustrated.

#### 2.1.2 3D printed housing for the first device

To install the board on the top tube of the bicycle, taking care not to cause any damage due to contact or external impacts and ensuring that power is supplied to the acquisition board, a 3D printed housing was designed and created. The housing consists of three parts, the frame, the bumper, and the base, as shown in Fig. 4 (parts 2, 3, 4, and 6, at center). In the base, both the acquisition board and the power bank (to ensure power supply to the board via a cable with a USB MINI A connector) are located, nevertheless, the 3D printed frame supports the PCB to be firmly attached to the base. The bumper serves to protect the acquisition board from possible impacts caused by the cyclist’s knees. A foam pad adjusts the power bank when inserted in the base. The base, the bumper, and the frame were printed using polylactic acid (PLA) due to the advantages it offers compared to other high polymer materials: it does not cause environmental contamination as it degrades into H_2_O and CO_2_, it is derived from crops instead of petroleum, it has good biocompatibility (non-poisonous and non-irritating, suitable for medical applications), and it does not experience a decline in plasticity and toughness over long-term use [45]. The parts were printed on a fused deposition 3D printer Artillery X1 with a 0.4 mm nozzle, a printing temperature of 210 °C, a filament diameter of 1.75 mm, a layer height of 0.2 mm, and 30% infill. The 3D-printed housing for the first device can be attached to the top tube of the bicycle using Velcro or any easily removable adhesive tape.

### 2.2 Second device

**Fig. 8:**
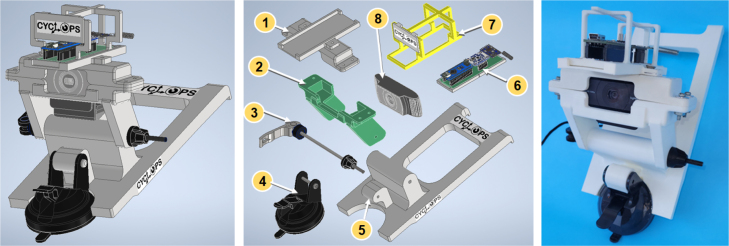
CAD design for the second device (left). The same device is in exploded view with numbered parts (center). (1) is the camera housing cap, (2) is the camera housing, (3) is the quick-release skewer, (4) is the suction cup, (5) is the base, (6) is the electronic acquisition board (including its frame), (7) is the bumper (including logo board), and (8) is the off-the-shelf RGB camera. The actual appearance of the second device after assembly is shown in the last image (right).

The second device consists of an electronic data acquisition board, a 3D-printed housing, and an off-the-shelf RGB camera, as seen in Fig. 8.

#### 2.2.1 Data acquisition electronic board

This board has the same dimensions, features, and components as the board located on the bicycle (Section 2.1.1). However, since it is positioned above the camera, which is inside the housing installed on the vehicle’s windshield, its components will be renamed to differentiate them from the same type of components assembled in the first device. The Arduino Nano is referred to as Microcontroller-V, the BNO055 is referred to as IMU-V, and the HC-12 transceiver is referred to as Transceiver-V.

#### 2.2.2 3D printed parts for the second device

A base was built to attach the components of the second device to the top border of the vehicle’s windshield through its salient claws. As can be seen in Fig. 8, the base (part #5) is linked to the suction cup (part #4) to adhere the base to the windshield. The base is also linked to a camera housing (part #2) that encloses the RGB camera in conjunction with the camera housing cap (part #1). The bumper (part #7 with a removable board including the CYCLOPS logo), protects the electronic board. All these parts were printed in PLA, using the same 3D printing characteristics as for the first device’s parts.

#### 2.2.3 Camera

The off-the-shelf camera used to acquire images was a Logitech C920 HD PRO WEBCAM. This is a 3 megapixels camera, auto-focus camera with maximum HD resolution images (1080p, 720p @ 30 fps), 78°in diagonal field of view (dFoV), and suited dimensions to attach to the car’s windshield (height: 43,3 mm, length: 94 mm, depth: 71 mm, weight: 162 g).

#### 2.2.4 Other parts

A suction cup (linked to the base through a rotational joint), was used to adhere the whole second device to the vehicle’s windshield. The camera housing can be manually leveled by tightening (or releasing) a quick-release skewer. These two parts are commercially available.

### 2.3 Software

The software, executed from a laptop was developed to receive, store, and correlate data obtained through IMUs (IMU-C and IMU-V), and the images captured by the camera. The software, called ‘VideoCapture’, was programmed in Python 3.8 and must be executed on Windows 10 or higher. The software allowed for the validation of the data acquisition system and its effective performance.

It can be highlighted the following advantages of CYCLOPS:

• It comprises an HD camera and two high-performance IMUs to acquire data at a low cost. So is a cheap prototype compared to off-the-shelf devices that comprise cameras and IMUs.
• CYCLOPS is an easy-to-implement, easy-to-adapt system, on any car or bicycle.
• It is an open software and open hardware system.
• Just by changing the housing design, a different camera can be used.

Based on a review done up to 05/2023, there are no devices with the same or similar purpose as CYCLOPS

## 3 Design files summary

In Table 1 it has been included both, CAD files for electronic assemblies and CAD files for structure assembly. CADs for structure include designs of PLA 3D printed pieces.

**Table 1:** Design files summary.

Design filename	File type	Open source license	Location of the file
First_Device.zip	CAD FILES	CC BY 4.0	https://doi.org/10.17632/3yx5y8b7tm.1
Second_Device.zip	CAD FILES	CC BY 4.0	https://doi.org/10.17632/3yx5y8b7tm.1
HalfFrame.stl	STL FILE	CC BY 4.0	https://doi.org/10.17632/3yx5y8b7tm.1
FirstDevice_Bumper .stl	STL FILE	CC BY 4.0	https://doi.org/10.17632/3yx5y8b7tm.1
FirstDevice_base.stl	STL FILE	CC BY 4.0	https://doi.org/10.17632/3yx5y8b7tm.1
CameraHousingCap .stl	STL FILE	CC BY 4.0	https://doi.org/10.17632/3yx5y8b7tm.1
CameraHousing.stl	STL FILE	CC BY 4.0	https://doi.org/10.17632/3yx5y8b7tm.1
SecondDevice_Base .stl	STL FILE	CC BY 4.0	https://doi.org/10.17632/3yx5y8b7tm.1
SecondDevice _Bumper.stl	STL FILE	CC BY 4.0	https://doi.org/10.17632/3yx5y8b7tm.1
PCB_inercial.dxf	dxf	CC BY 4.0	https://doi.org/10.17632/3yx5y8b7tm.1
PCB_PCB_inercial. json	json	CC BY 4.0	https://doi.org/10.17632/3yx5y8b7tm.1
Drill_PTH_ Through.DRL	CAD FILE	CC BY 4.0	https://doi.org/10.17632/3yx5y8b7tm.1
Drill_PTH_ Through_Via.DRL	CAD FILE	CC BY 4.0	https://doi.org/10.17632/3yx5y8b7tm.1
Gerber_Bottom Layer.GBL	CAD FILE	CC BY 4.0	https://doi.org/10.17632/3yx5y8b7tm.1
Gerber_BottomS ilkscreenLayer .GBO	CAD FILE	CC BY 4.0	https://doi.org/10.17632/3yx5y8b7tm.1
Gerber_BottomSo lderMaskLayer .GBS	CAD FILE	CC BY 4.0	https://doi.org/10.17632/3yx5y8b7tm.1
Gerber_Board OutlineLayer .GKO	CAD FILE	CC BY 4.0	https://doi.org/10.17632/3yx5y8b7tm.1
Gerber_TopLayer .GTL	CAD FILE	CC BY 4.0	https://doi.org/10.17632/3yx5y8b7tm.1
Gerber_Top SilkscreenLayer .GTO	CAD FILE	CC BY 4.0	https://doi.org/10.17632/3yx5y8b7tm.1
Gerber_TopSolder MaskLayer .GTS	CAD FILE	CC BY 4.0	https://doi.org/10.17632/3yx5y8b7tm.1

## 4 Bill of materials summary

*All the required components for the fabrication of the device are summarized in* Table 2 *.*

**Table 2:** Bill of materials.

Designator	Component	Number	Cost per unit -($US)	Total cost - ($US)	Source of materials	Material type
Imu	BNO055	2	34,95	69,9	https://bit.ly/3J8Jfhw	Composite
Microcontroller	Arduino nano	2	24,9	49,8	http://bit.ly/41XE50C	Composite
Transceiver	HC12	2	8,99	17,98	https://bit.ly/3YvjfCU	Composite
Resistor	Resistor	2	0,04	0,08	https://bit.ly/3T61ZmD	Composite
Led	Led diode	2	0,05	0,1	https://bit.ly/3JpAIZ3	Composite
Headers	Pin headers female	96	1,68	3,36	https://bit.ly/3JD0eud	Composite
Power bank	Power bank	1	21,5	65,95	http://bit.ly/3l4rqbu	(Off-the-shelf device)
pcb	Printed circuit board	2	20	50	(Own development)	FR - 4
Logitech C920 Webcam 1080 HD	Logitech C920 Webcam 1080 HD	1	64	64	https://bit.ly/3ZzzuAg	(Off-the-shelf device)
USB cable	USB cable type A	1	7,14	7,14	https://bit.ly/3kZwApn	Non specified
Support frame	3D printed Support frame for PCB	2	4	8	(Own development)	PLA
Base (first device)	3D printed base – first device	1	10	10	(Own development)	PLA
Bumper	3D printed bumper	2	5	10	(Own development)	PLA
Camera housing cap	3D printed camera housing cap	1	10	10	(Own development)	PLA
Camera housing	3D printed camera housing	1	10	10	(Own development)	PLA
Base (second device)	3D printed base – second device	1	15	15	(Own development)	PLA
Foam pad	Neoprene foam pad	1	14,99	14,99	https://amzn.to/3pv4hBe	Foam
Suction cup	Suction cup for attaching the housing to the vehicle	1	11,99	11,99	https://amzn.to/40VZ1mW	Rubber
Quick release skewer	Quick release skewer	1	14,99	14,99	https://amzn.to/3oYcDka	Steel

## 5 Build instructions

Before starting the assembly process of the devices, it will be necessary to manufacture two electronic data acquisition boards (one for each device), using the electronic design files available for this purpose in Mendeley and the list of parts in Table 2. In addition, the following parts, corresponding to the housings of both devices must be printed in 3D: cameraHousing.stl; cameraHousingCap.stl; firstDevice_base.stl; firstDevice_Bumper.stl; halfFrame.stl (print 4 of these); secondDevice_Base.stl; secondDevice_Bumper.stl (see Fig. 9, Fig. 10).

### 5.1 Build instructions for the first device

**Fig. 9:**
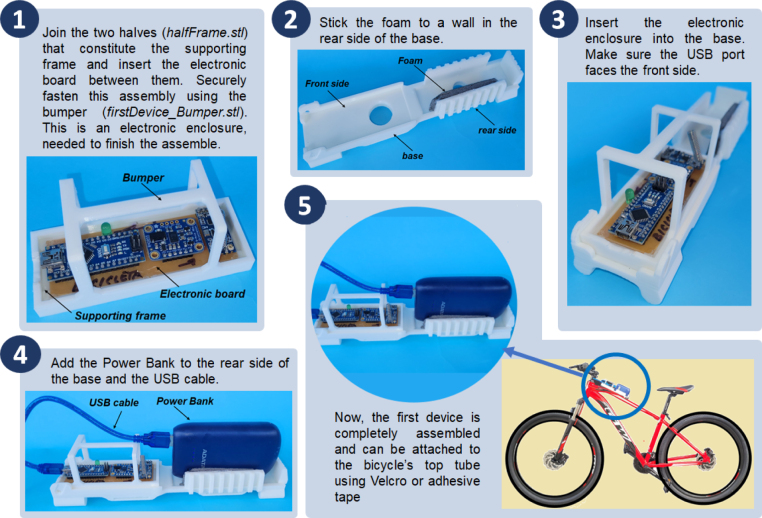
Build instructions for the first device, attachable to the bicycle’s top tube.

### 5.2 Build instructions for the second device

**Fig. 10:**
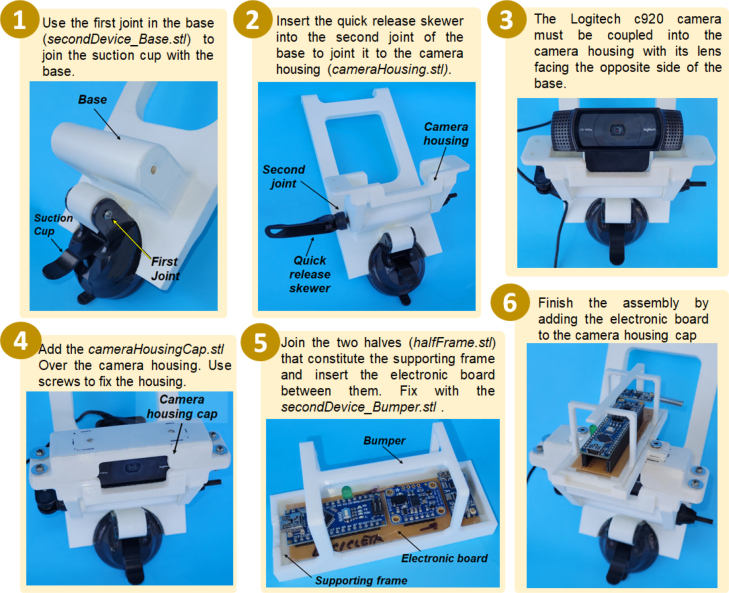
Build instructions for the second device, attachable to the vehicle’s windshield.

## 6 Operation instructions

After assembling all the hardware devices comprising the system, the next step involves configuring the inertial measurement units, programming the microcontrollers, and executing the acquisition software on the laptop. To accomplish this, a laptop computer with two USB 3.0 ports is required (one for the acquisition card installed on the camera and another for the camera itself). The laptop should be running Windows 10 or a higher operating system version (Linux is a viable alternative). Additionally, Python 3.0 (or a higher version) must be installed alongside the “Python serial library”. The Arduino IDE with its “SoftwareSerial” library installed is also necessary for programming the microcontrollers. Once all the prerequisites are fulfilled, the following procedure should be followed. Besides, make sure that OpenCV2 libraries for connecting the RGB camera are installed in your laptop. The BNO055 libraries are also needed (Adafruit BNO055 Library and Adafruit sensor Library) to correctly configure the IMUs via the microcontrollers.

### 6.1 Calibrating the IMUs

The IMUs are part of two devices (the first device on the bicycle and the second device in the vehicle), and therefore, both sensors must be calibrated separately. Due to this, a different ID will be used for each IMU in the calibration program. The calibration code called “Cal_imu_bicycle.ino” will then be loaded into the Microcontroller-C of the first device. Similarly, the calibration program “Cal_imu_Car.ino” must be loaded into the Microcontroller-V of the second device.

To achieve reliable calibration, the following steps should be followed for both IMU-C and IMU-V:

• Calibrate the accelerometer by tilting the IMU 45°and −45°, sequentially in the directions of the +X, −X, +Y, −Y, +Z, −Z axes.
• Calibrate the gyroscope by keeping it still for 2 s on a flat horizontal surface.
• Calibrate the magnetometer by moving it in random positions until the Arduino serial monitor shows a value of 3, indicating that the calibration values have been reached

Once the calibration process is complete, the data acquisition programs must be loaded into both the Microcontroller-C (program named “Cyclops_Bicycle_Side.ino”) and the Microcontroller-V (program named “ Cyclops_Car_Side.ino ”). The first program allows obtaining the acceleration and orientation data from IMU-C and sending it to the second device. The second program allows obtaining the acceleration and orientation data from IMU-V, receiving the data from IMU-C, and sending them (together) to a computer via USB cable for storage.

The software files required for the configuration and proper operation of CYCLOPS are available for download, as presented in Table 3.

**Table 3:** Software files summary.

Software file name	File type	Open source license	Location of the file
Adafruit BNO055 Library	Lib	MIT Licence	https://github.com/adafruit/Adafruit_BNO055
Adafruit sensor Library	Lib	MIT Licence	https://github.com/adafruit/Adafruit_Sensor
OpenCV2 libraries	Lib	BSD license	https://opencv.org/releases/page/5/
Python serial library	Lib	BSD-3-Clause	https://github.com/pyserial/pyserial
VideoCapture.rar	rar/exe	CC BY 4.0	https://doi.org/10.17632/3yx5y8b7tm.1
Cal_imu_bicycle.ino	Ino	CC BY 4.0	https://doi.org/10.17632/3yx5y8b7tm.1
Cal_imu_car.ino	Ino	CC BY 4.0	https://doi.org/10.17632/3yx5y8b7tm.1
Cyclops_Bicycle _Side.ino	Ino	CC BY 4.0	https://doi.org/10.17632/3yx5y8b7tm.1
Cyclops_Car _Side.ino	Ino	CC BY 4.0	https://doi.org/10.17632/3yx5y8b7tm.1

### 6.2 Storing images and inertial measurement data

On the computer (typically a laptop), the software responsible for storing both the images and data from the two IMUs must be installed. This software, called “VideoCapture”, is an executable developed specifically for the proper functioning of the acquisition system. The computer must have Windows 10 or later operating system and have two USB ports (one to connect the camera and another to connect the vehicle’s data acquisition electronic board), it is also necessary to have OpenCV libraries installed in the computer.

To ensure proper functioning of the software, the “VideoCapture” file must be copied to the root of the computer’s drive. Then, it must be determined which port the camera has been connected to and which port the acquisition card of the second device has been connected to.

The parameters of the software must be configured in the “config.ini” file located at the path: \VideoCapture \dist \VideoCapture \config.ini. The parameters to be configured are as follows:

• VideoCamera = 1 to use the camera connected to the USB port.
• Image resolution in pixels: it is suggested to work with a maximum Full HD resolution.
• IMUserialport = Serial port assigned by the PC to the acquisition card.
• Baudrate = Always set to 9600 baud.
• Disable IMU reading: 1 to NOT read IMU data, 0 to use IMU.
• TestingTimeInMinutes = Total acquisition time specified in minutes.
• FramesDelay = Sample time (or delay time) in seconds between frames.
• QData = Expected amount of data in the reading of the hardware module connected to the computer for testing.

To start the acquisition process, the “VideoCapture.exe” file must be executed. The program will receive the calibration data from the IMUs and then display a message enabling the start of the acquisition process. Then, the user must click the “enter” key, and from that moment a timer will show the elapsed acquisition time on the screen (the acquisition can be stopped at any time using the “ctrl+c” keys combination).

When the acquisition time has ended, the user can find the images and their corresponding data stored in the computer’s drive at: \VideoCapture \dist \VideoCapture \VideoFrames.

## 7 Validation and characterization

CYCLOPS has been developed to meet the need for a cyclist data acquisition system that enables capturing both the cyclist’s moving images and their inertial values (accelerations and orientations in three axes) and correlating them as they are stored. With its development, CYCLOPS contributes to the creation of a database that will allow training neural networks to utilize this data for, predicting – among other things – the movement intentions of cyclists from an autonomous vehicle and reacting accordingly.

**Fig. 11:**
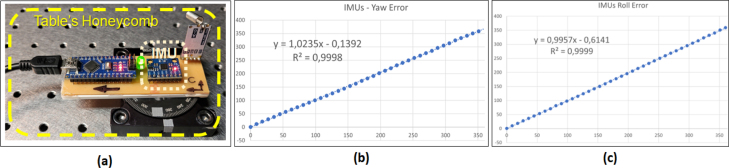
IMU’s characterization. In (a) the electronic acquisition board has fixed to the goniometer with the IMU’s axis aligned with the goniometer’s rotation axis. In (b) the proportion of variance between real rotated Yaw angle and IMU’s Yaw angle was calculated as an R-square value and the best fitted equation has been obtained. The same is presented in (c) for the IMU’s Roll angle. As can be seen in the graphs, the R2 value is almost 1, which can be interpreted as highly accurate performance of the IMU.

After assembling the two constituent devices of CYCLOPS, the IMUs were characterized using equipment from the Vision and Photonics Laboratory at the Instituto Tecnológico Metropolitano de Medellín https://www.itm.edu.co/investigacion/laboratorios/optica-fotonica-y-vision-artificial/ Opening from Chrome. To achieve this, the following procedure was conducted:

• The IMUs were characterized on a Kinetic Systems 5300 Series optical table https://kineticsystems.com/products/optical-tables/ultimate-grade-5300-series-optical-tables/, which incorporates quad-tuned dampers in each corner to ensure maximum stability and minimal response to vibration disturbances.
• Each of the IMUs was calibrated according to the procedure outlined in Section 6.
• Each of the electronic acquisition boards (both for the first and second device) was placed horizontally with the IMU’s central axis, aligned with the central axis of a goniometer to measure the Yaw value (around the Z-axis), as shown in Fig. 11a.
• Controlled angular movements of the IMU were performed around the goniometer axis, at nine-degree (9°) intervals, which were randomly established until complete 360°(40 samples).
• The actual rotated value was plotted against the value outputted by the IMU, and the R2 value and fitting equation were obtained.
• The procedure was repeated with the board in a vertical position to read the Roll value (around the X-axis).

The graphs resulting from the characterization can be seen in Figs. 11b and 11c.

**Fig. 12:**
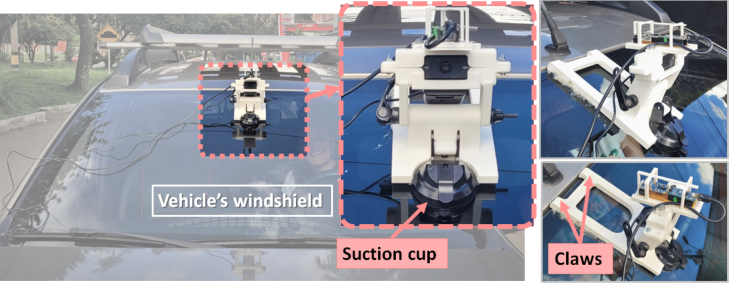
Installation of the second device onto the vehicle’s windshield using the suction cup and claws. The acquisition electronic board is positioned directly above the camera.

**Fig. 13:**
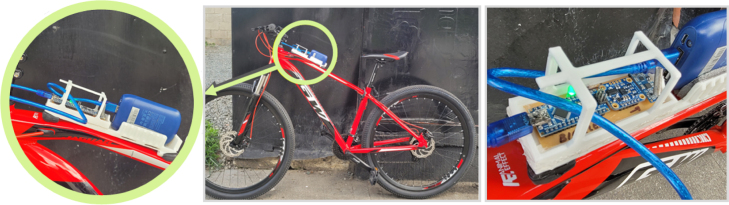
The first device was installed over the bicycle’s top tube (left). Velcro belts, adhesive tape or -as did in this test) glue can be used to fix the housing base to the tube (center). The USB wires were connected from the power bank to the electronic board before beginning the acquisition process.

Subsequently, the validation of the entire system’s functionality was carried out. For this purpose, the first device was installed on a bicycle, and the second device on a vehicle (see Fig. 12, Fig. 13). Subsequently, both sensors were synchronized by powering the electronic data acquisition cards, as depicted in Fig. 11. From that moment, the software configuration was performed, selecting the total acquisition time (in minutes) and the sampling time (in seconds), as specified in Section 6.2 of the “config.ini” file of the “VideoCapture” software.

**Fig. 14:**
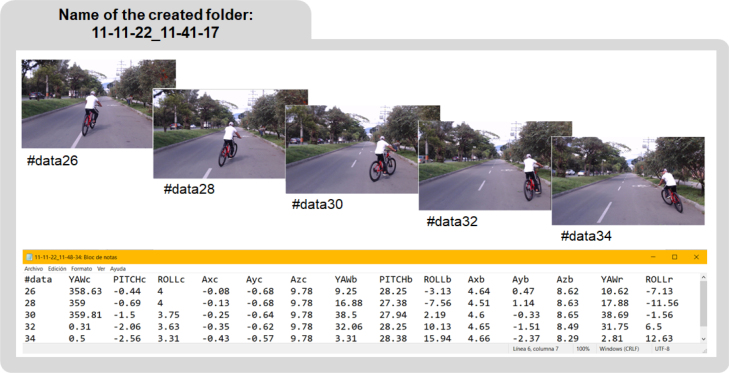
The RGB images acquired at each sampling of the process are correlated with the data in each row of the file generated by the CYCLOPS system. (B) The number of rows in the file corresponds to the ratio between the total acquisition time and the sampling period. Each row contains 13 data points. The first data point corresponds to the data number. The following six data points correspond to the orientation angles and accelerations of the vehicle (YAWv, PITCHv, ROLLv, AXv, AYv, AZv). The last six data points correspond to the orientation angles and accelerations of the cyclist (YAWc, PITCHc, ROLLc, AXc, AYc, AZc).

Having completed the previous steps, the software initiated the process of data acquisition and storage. Once the total acquisition time elapsed, a folder was generated within the path: \VideoCapture \dist \VideoCapture \VideoFrames. The folder contained the acquired images and a *.txt file with the corresponding inertial data. For each image, the *.txt file had a row of twelve data organized as follows: the first data (#data) indicated the row number of the data (as many rows of data as acquired images). Subsequently, there were three vehicle orientation data for the X, Y, and Z axes, denoted as YAWv, PITCHv, and ROLLv, respectively. In the same row, the following three data represented the vehicle accelerations along the X, Y, and Z axes, denoted as AXv, AYv, and AZv, respectively. The three cyclist orientation data, YAWc, PITCHc, and ROLLc, followed by the three cyclist acceleration data, AXv, AYv, and AZv, completed the twelve data points in each row (see Fig. 14).

• The tests demonstrated that the developed software ensured that the system generates corresponding images and inertial data with the same timestamp.
• The quality of the obtained data directly depended on the proper calibration of the IMUs.
• When the road has many irregularities, there is a risk of obtaining inaccurate data if the sampling period is too low (less than 0.2 s). Additionally, constant vibration can lead to cable disconnection in the installed devices, resulting in errors in the acquired data.
• The acquired data will be of great utility in creating a database that will be used for training neural networks aimed at determining the orientation and inclination of cyclists on the road by an autonomous vehicle’s driving system.

CYCLOPS has been developed to acquire data in urban environments, not in rural settings. Therefore, the experiments shown in this work, validating its performance, have been carried out on paved roads with few irregularities. However, since urban roads can also present irregularities that affect data quality, the IMU (BNO055) was chosen among the different options on the market based on the manufacturer’s information. As described by [44], the BNO055 IMU uses sensor fusion algorithms that include filters to estimate the internal state of the IMU in the presence of uncertainties. This is why the data acquired on urban roads with CYCLOPS is minimally affected by irregularities in paved roads.

Some authors have used the same IMU (BNO055) or similar IMUs developed under MEMS technology to determine the effect of vibrations on vehicles on roads with irregularities. In [46], the effect of vibrations on MEMS accelerometers, located inside a vehicle, is investigated. The results indicate that the accuracy of inertial measurements of MEMS accelerometers can be affected by ground vibrations as their frequencies approach the resonant frequencies of the accelerometer components, increasing the amplitude of the measured signal and, therefore, distorting the real acceleration data. If the ground irregularities are so large that the generated vibrations range between 0.5 g and 2.5 g, the optimum operating range of the MEMS accelerometer is exceeded, so measurements become inaccurate. The above implies that acquiring inertial measurement data on irregular terrain would require a detailed analysis of the vibration environment to be carried out beforehand and systems to be designed with IMUs that can withstand the expected conditions, as well as applying advanced filtering techniques to isolate the signals of interest from unwanted vibrations. In this latter aspect, in [47], the authors use a combination of adaptive least mean squares (LMS) filters and low-pass finite impulse response (FIR) filters to deal with the vibrations that affect the signals from a BNO055 IMU that has been installed on the dashboard of a vehicle. The experiments presented by the authors demonstrate that the proposed filter combination offers a better signal-to-noise ratio (SNR) and noise attenuation ratio (ATT) compared to filtering systems that include MEMS IMUs. This strategy, according to the authors, allows improving the quality of the acquired data affected by the vibrations generated by gravel roads, bumpy roads, sewer covers, and even road marks.

The studies presented are oriented to vehicles, therefore, considering that bicycles are more prone to be affected by the irregularities of the terrain, it will be mandatory to consider the recommendations of adding filtering algorithms to the data if a dataset with good quality data is expected.

## CRediT authorship contribution statement

**Mauricio Arias-Correa:** Writing – original draft, Visualization, Methodology, Investigation, Conceptualization. **Sebastián Robledo:** Writing – original draft, Validation, Software. **Mateo Londoño:** Writing – original draft, Validation, Software. **Johnatan Bañol:** Validation, Software. **Carlos Madrigal-González:** Writing – review & editing, Supervision. **John R. Ballesteros:** Writing – review & editing, Supervision. **John W. Branch-Bedoya:** Supervision, Resources, Funding acquisition.

## Declaration of competing interest

The authors declare that they have no known competing financial interests or personal relationships that could have appeared to influence the work reported in this paper.
